# The Effect of Cannabidiol on Subjective Responses to Endurance Exercise: A Randomised Controlled Trial

**DOI:** 10.1186/s40798-024-00727-3

**Published:** 2024-05-23

**Authors:** Danielle McCartney, Christopher Irwin, Zeeta Bawa, Blake Palmer, Ayshe Sahinovic, Nathan Delang, Gregory R. Cox, Ben Desbrow, Namson S. Lau, Iain S. McGregor

**Affiliations:** 1 Lambert Initiative for Cannabinoid Therapeutics, The University of Sydney, Sydney, NSW Australia; 2 Brain and Mind Centre, The University of Sydney, Sydney, NSW Australia; 3 School of Psychology, Faculty of Science, The University of Sydney, Sydney, NSW Australia; 4 School of Health Sciences and Social Work, Griffith University, Gold Coast, QLD Australia; 5 Menzies Health Institute Queensland, Griffith University, Gold Coast, QLD Australia; 6 Sydney Pharmacy School, The University of Sydney, Sydney, NSW Australia; 7 Faculty of Health Sciences and Medicine, Bond University, Gold Coast, QLD Australia; 8 The Boden Initiative, Charles Perkins Centre, The University of Sydney, Sydney, NSW Australia; 9 The University of Sydney, 94 Mallett Street, Camperdown, NSW 2050 Australia

## Abstract

**Background:**

Exercise is known to improve health. However, it can be unpleasant, often inducing negative feelings, or ‘affect’. Cannabidiol (CBD), a non-intoxicating constituent of the cannabis plant, has been reported to enhance the subjective experience of exercise; specifically, in trained individuals performing fixed-intensity endurance activity. Here, we investigated the effects of CBD on subjective responses to exercise under more ecologically valid conditions; namely, in recreationally active individuals performing self-paced endurance activity.

**Methods:**

A randomised, double-blind, placebo-controlled, crossover trial was conducted at Griffith University between July 17 and August 28, 2023. Griffith University students studying sports nutrition were invited to take part, with eligible volunteers ≥ 18 years of age and able to perform endurance exercise. Participants ingested placebo or 150 mg CBD in two soft-gel capsules 90 min before completing a self-paced 25-lap (10 km) run around an outdoor athletics track (400 m, synthetic). The primary outcomes were *affective valence during exercise*, assessed on completion of laps 6, 12, 18 and 24 using the ‘Feelings Scale’, and *positive and negative affect*, assessed at baseline, pre-run and post-run using the ‘Positive and Negative Affect Schedule’. Exercise enjoyment, motivation and self-efficacy, the core features of the ‘runner’s high’ (i.e., euphoria, pain, anxiety, sedation), perceived exertion and run time were also assessed.

**Results:**

Fifty-two participants were randomised and 51 were included in the final sample (*n* = 22 female; 22 [21–25] years). Exercise induced negative affect (i.e., at the time of undertaking) and increased pain. CBD did not counteract either response. In fact, CBD had no significant effects on any of the outcomes measured. In contrast, exercise, *once completed*, increased positive affect, and decreased negative affect and anxiety.

**Conclusions:**

CBD (150 mg, oral) does not appear to enhance the subjective experience of self-paced endurance exercise in recreationally active individuals. Nor, however, does it appear to compromise it. These findings suggest that CBD use is safe under exercise conditions and unlikely to impede physical activity participation. Our study also reaffirms the powerful mood-enhancing effects of exercise.

**Trial Registration:**

Registered with the Australian New Zealand Clinical Trials Registry (www.anzctr.org.au) on May 31, 2023 (Trial ID: ACTRN12623000593639).

**Supplementary Information:**

The online version contains supplementary material available at 10.1186/s40798-024-00727-3.

## Key points

• Does cannabidiol (CBD), a non-intoxicating constituent of *Cannabis sativa* L., enhance the ‘subjective experience’ of exercise?

• In this randomised, double-blind, placebo-controlled, crossover trial of 51 recreationally active individuals, CBD (150 mg) did not increase positive (or decrease negative) feelings during or following a self-paced 10 km run.

• CBD (150 mg) does not appear to alter subjective responses to self-paced endurance exercise in recreationally active individuals.

**Supplementary Information:**

The online version contains supplementary material available at 10.1186/s40798-024-00727-3.

## Introduction

Exercise is known to improve physical and mental health [1]. However, it can be unpleasant, often inducing physical discomfort, pain, fatigue, and negative feelings, or ‘affect’ [2].

Cannabis use has been reported to enhance the subjective experience of exercise (at least in habitual cannabis users). Indeed, most of the physically active cannabis users surveyed in two recent studies [3, 4] endorsed using cannabis prior to exercise – often to increase exercise enjoyment. Two recent interventional studies [5, 6] likewise found that *ad libitum* cannabis use increased positive affect and enjoyment, and decreased negative affect and pain, during running exercise compared to a ‘no cannabis’ control.

The subjective effects of cannabis use (including those observed in the studies described above [3–6]) can largely be attributed to *Δ*^*9*^*-tetrahydrocannabinol* (THC) [7]. However, *cannabidiol* (CBD), a non-intoxicating constituent of the cannabis plant [8–10] that can be purchased over-the-counter in many countries (i.e., in ‘nutraceutical products’) [11, 12], is not without mood-altering potential. Indeed, CBD appears to modulate receptors in the central nervous system (CNS), including some of those related to mood regulation (e.g., the serotonin 1 A receptor [5-HT_1A_] [13, 14]). (Note, however, that it has an entirely different set of pharmacological actions compared to THC [7]). CBD has also demonstrated efficacy in treating affective disorders (e.g., anxiety [15–21], preclinical models of depression [22–28]) and been reported, albeit inconsistently [29], to increase blood concentrations of anandamide [30, 31], an endogenous cannabinoid that appears to contribute to the ‘runner’s high’ (i.e., ‘pleasant’ feeling sometimes experienced during endurance exercise) [32].

Two recent studies have investigated the effects of CBD on subjective responses to exercise. Gibson et al. [6] conducted a semi-randomised, controlled, crossover trial in 11 “highly active” cannabis users. It showed that CBD-dominant cannabis (20% CBD; 1% THC), inhaled *ad libitum*, increased positive affect and enjoyment during a 30-minute fixed-intensity treadmill run compared to a ‘no cannabis’ control. Meanwhile, Sahinovic et al. [29] conducted a randomised, double-blind, placebo-controlled, crossover (pilot) trial in nine endurance-trained males (VO_2max_: 57.4 mL/kg/min). It showed that CBD (300 mg, oral) increased positive affect during a 60-minute fixed-intensity (70% VO_2max_) treadmill run. These preliminary findings suggest that CBD may enhance the subjective experience of exercise. However, further research is required to confirm as such and determine whether this effect is sustained under more ecologically valid conditions; namely, when using lower oral doses of CBD, consistent with those available over-the-counter (i.e., ≤ 150 mg in Australia) [11, 12], and in recreationally active individuals performing self-paced endurance activity.

With this in mind, the overall aim of the current study was to investigate the effects of CBD (150 mg, oral) on subjective responses to self-paced endurance exercise in recreationally active individuals. We hypothesised that CBD would enhance the subjective experience of exercise as, primarily, evidenced by an increase in positive (or decrease in negative) affect during and following activity.

## Methods

### Study Design

A randomised, double-blind, placebo-controlled, crossover, clinical trial was conducted at Griffith University (Gold Coast Campus, Southport, QLD). The trial was approved by Griffith University’s Human Research Ethics Committee (GU Ref No: 2023/253), conducted in accordance with the standards of ethics outlined in the Declaration of Helsinki, and registered prospectively with the Australian New Zealand Clinical Trials Registry (Trial ID: ACTRN12623000593639). All participants provided written informed consent prior to enrolment (i.e., any information/data being obtained).

### Participants

Griffith University students enrolled in ‘3138AHS Exercise Sports Nutrition’ in 2023 were invited to participate. Eligible volunteers were: (1) ≥ 18 years of age; (2) proficient in English; and (3) able to perform endurance exercise. The final criterion was assessed using the ‘Physical Activity Readiness Questionnaire for Everyone’ [33]. Volunteers who answered ‘no’ to all of the questions in Part 1 or Part 2, or who answered ‘yes’, but were later cleared by the trial physician (N.L.) following further evaluation, were considered suitable to participate.

The following exclusion criteria applied: (1) a self-reported history of allergic reaction to cannabis or cannabinoid-containing products; (2) a self-reported history of liver disease or renal disease; (3) a self-reported or physician-suspected history of drug/alcohol dependence; (4) self-reported or physician-suspected suicidal ideation; (5) regular (i.e., > 2/week) use of cannabis or CBD; (6) unwilling to adhere to trial procedures; and (7) pregnant, lactating or trying to conceive.

### Randomisation

Participants were randomised (1:1) to one of two possible treatment orders at the beginning of the first treatment session. Specifically, they were assigned a unique identification (ID) code (by the principal investigator, D.M.) that was linked to a treatment order via a pre-populated randomisation schedule. The schedule was generated in 12 balanced blocks of 10 by an independent statistician using an online random number generator (www.sealedenvelope.com/). Treatment allocation was then concealed using ‘numbered containers’ (i.e., single-dose sachets carrying participant ID codes and treatment session numbers).

### Blinding

Only the aforementioned statistician, one independent researcher, and the company that packaged and labelled the treatments could access the randomisation schedule, none of whom had any contact with participants or further involvement in the trial.

### Treatments

The treatments were purchased from Avecho Biotechnology Limited (Clayton, VIC), manufactured (Catalent Pharma Solutions, St. Petersburg, FL) and packaged (Central Pharmacy Logistics, Coburg North, VIC) at GMP-licenced facilities, stored at Griffith University’s Clinical Trials Unit, prescribed by the trial physician (N.L.) (under the Clinical Trials Notification scheme), and administered (by the trial pharmacist, Z.B. and another investigator, I.S.M.) at the Griffith University Athletics Track.

#### Intervention

The intervention was encapsulated CBD. Each (soft-gel) capsule contained 75 mg of pure, synthetic -(-) CBD and 75 mg of Tocopherol Phosphate Mixture® (TPM) in medium-chain triglyceride oil (350 mg) (as confirmed on the Certificate of Analysis). TPM is a proprietary blend of Vitamin E phosphates that has been shown to enhance the oral bioavailability of lipophilic substances [34].

##### Dose

150 mg CBD (i.e., two soft-gel capsules) was administered via oral ingestion.

#### Control

The control was a placebo. It was identical to the intervention but did not contain any CBD.

### Treatment Sessions

Participants completed two treatment sessions at the Griffith University Athletics Track. The sessions were held on August 21 and August 28, 2023 (i.e., as “mass participation” events) with individuals receiving CBD on one occasion and placebo on the other. Indeed, 150 mg CBD appears to washout [35], and exercise-induced muscle soreness appears to subside [36], within 7 days. Participants provided demographic information (i.e., via the completion of an online questionnaire) in the weeks preceding the first treatment session.

#### Standardisation Procedures

Prior to each session, participants were instructed to: (1) avoid using alcohol, cannabis, and CBD (≥ 24 h); (2) avoid exercise (≥ 12 h); (3) spend ≥ 6 h in bed overnight; (4) fast overnight (≥ 6 h); and, on waking, (5) consume 500 mL water and (6) no more than their usual morning ‘dose’ of caffeine (± milk and sugar) ≥ 1 h prior to arrival. No further (pre-session) standardisation procedures were employed.

#### Experimental Procedures

Participants arrived at the facility in a semi-fasted state (i.e., having only consumed their usual morning dose of caffeine) between ∼ 7:00–8:00 AM and were asked whether: (1) they had complied with each of the standardisation procedures; and (2) their health status or medication use had changed since last contact. They then completed a breath alcohol test (Alcolizer LE5, Alcolizer Technology), a urine hydration test (IC-Pen-Urine SG Digital Refractometer, ATAGO), and a baseline questionnaire before (provided they were still eligible to participate, had avoided using cannabis and CBD, and were neither intoxicated nor hungover) consuming their assigned treatment.

Following treatment administration, participants were offered a pre-packaged breakfast meal (i.e., commercial box containing cereal, milk, stewed fruit, fruit juice, and a muesli bar) (LePack Accommodation Supplies Australia, Southport, QLD) and fresh fruit. They were instructed to consume as much or as little as they liked at their first treatment session and to replicate this dietary behaviour at their second.

Participants completed a pre-run questionnaire 75 min post-treatment and commenced a self-paced 25-lap (10 km) run around a standard outdoor athletics track (400 m, synthetic) 90 min post-treatment, running to the same self-selected ‘goal’ (i.e., *as fast as possible*, *as fast as comfortably possible*, or *at a tolerable pace*) and in the same ‘social context’ (i.e., *predominantly alone* or *predominantly with one partner*) on each occasion. Individuals: (1) were instructed to *run* (i.e., limit walking); (2) did not receive any encouragement or feedback on time elapsed; and (3) were prohibited from listening to music, eating, and drinking throughout exercise. They also wore ‘bibs’ carrying the numbers 1 to 25 (where 6, 12, 18 and 24 were highlighted). Participants crossed one number off per lap (under the supervision of research staff) using a marker they carried and gave verbal responses to questions on completion of laps 6, 12, 18 and 24 (i.e., ‘on-the-go’).

Participants completed a post-run questionnaire ∼ 15 min after ceasing exercise and were asked whether they had experienced any “unfavourable signs or symptoms” (i.e., adverse events) before leaving the facility.

### Outcomes

#### Primary Outcomes

- Affective valence during exercise assessed on completion of laps 6, 12, 18 and 24 using the 11-point Feelings Scale (FS) [37], where − 5 = “feeling very bad” and + 5 = “feeling very good”.
- Positive and negative affect assessed at baseline, pre-run and post-run using the 20-item Positive and Negative Affect Schedule (PANAS) [38], where higher scores (range: 10–50) represent greater positive and negative affect.

#### Secondary Outcomes

- Exercise enjoyment assessed post-run using the 18-item Physical Activity Enjoyment Scale [39], where higher scores (range: 18–126) represent greater enjoyment.
- Euphoria, pain, anxiety, and sedation (i.e., the core features of the ‘runner’s high’ [32]) assessed at baseline, pre-run and post-run using 100 mm visual analog scales (VASs), where zero = “not at all” and 100 = “extremely”.
- Exercise motivation and self-efficacy assessed post-run using 100 mm VASs (‘*how motivated are you right now to run three times/week for 25 minutes?’* and ‘*how confident are you right now that you could run three times/week for 25 minutes?’*), where zero = “not at all” and 100 = “extremely”.
- Perceived exertion during exercise assessed on completion of laps 6, 12, 18 and 24 using the 15-point Borg Scale [40], where 6 = “no exertion” and 20 = “maximal exertion”.
- Run time, calculated by subtracting each participant’s start time (on the minute) from their finish time (to the current minute).

### Data Analysis

‘Single-point’ continuous variables were analysed using random-intercept linear mixed-effects models that included Treatment (categorical: CBD, Placebo) as a fixed effect and Participant as a random effect. Sex (categorical: Male, Female) and/or Run (i.e., trial order) (categorical: Run 1, Run 2) were also included as fixed effects if they reduced the Akaike Information Criterion (AIC) value of the model. If the residuals were non-normally distributed (Shapiro-Wilk test, *p* < 0.05) and/or heteroscedastic (Levene test, *p* < 0.05), the dependent variable was square-root transformed and re-analysed (and if unimproved, log-transformed). If neither transformation was curative, a gamma generalised linear mixed-effects model was substituted. This model used an ‘identity’ link unless the data were skewed – in which case, a ‘log’ link was trialled. If an appropriate gamma model could not be generated (e.g., it failed to converge and/or demonstrated singular fit) the ‘best’ of those described above (i.e., simplest model violating the fewest assumptions) was utilised.

‘Serial’ continuous variables were analysed using the same approach, except the models: (1) had random-intercepts *and* slopes; and (2) included Time (categorical: Baseline, Pre-Run, Post-Run) and the Treatment × Time interaction as fixed effects.

Ordinal variables (i.e., affective valence, perceived exertion, sleep quality) were analysed using cumulative link mixed-effects models. These models had random-intercepts and slopes, and included Treatment, Lap (continuous) and the Treatment × Lap interaction as fixed effects (as appropriate). Sex, Run (i.e., trial order) and/or Lap^2^ were also included as fixed effects if they reduced the AIC value of the model.

Two-sided, Dunn–Šidák-corrected post-hoc comparisons were used to compare estimated marginal means if a significant main or interaction effect was observed. Uncorrected a priori planned post-hoc comparisons of FS ratings on placebo and CBD at the 6-, 12-, 18- and 24-lap time points were also performed. Statistical significance was accepted as *p* < 0.05.

All statistical analyses were performed in R version 4.2.0 [41] using the following packages: ‘lme4’ (*lmer* and *glmer* functions) [42], ‘lmerTest’ [43], ‘ordinal’ (*clmm* function) [44], ‘RVAideMemoire’ (*Anova.clmm* function) [45], ‘emmeans’ (*emmeans* function) [46], ‘Car’ (*Anova*, *qqp* and *LeveneTest* functions) [47], ‘MuMIn’ (*AICc* function) [48] and ‘ggplot2’ (*ggplot* function) [49]. All values presented in text are estimated marginal mean (95% confidence interval [CI]), unless otherwise stated.

#### Sample Size

Sahinovic et al. [29] found that CBD (300 mg, oral) increased FS ratings during a 60-minute fixed-intensity (70% VO_2max_) treadmill run compared to placebo (Cohen’s *d*_z_ ≈ 0.70). Using a power (1-β) of 0.95, a two-sided α of 0.05, and a more conservative Cohen’s *d*_z_ of 0.40, we predicted a priori that 84 participants would be required to detect a significant effect of CBD on affective valance at the 6-, 12- 18- and 24-lap time points.

## Results

### Participant Recruitment and Retention

**Fig. 1:**
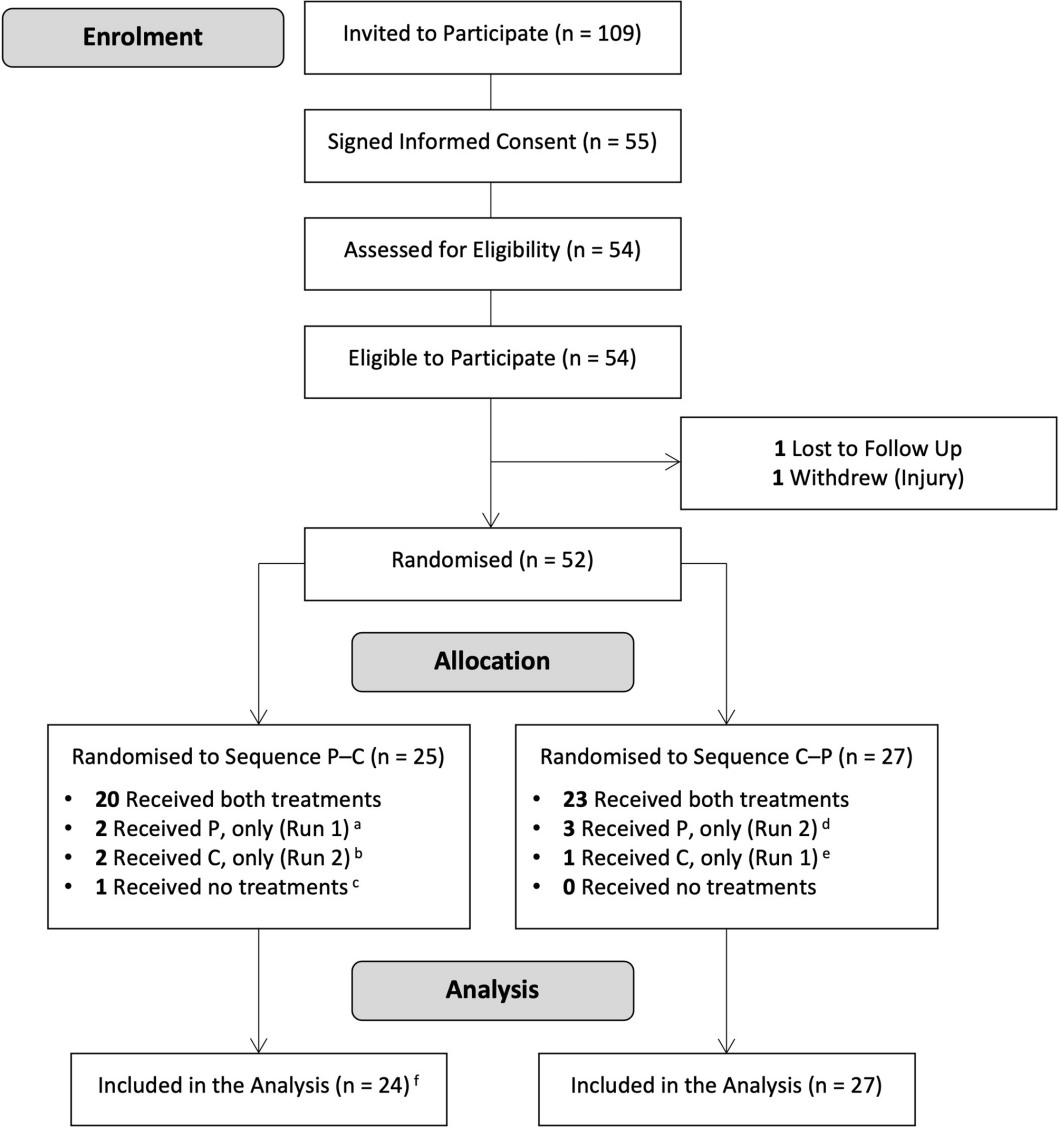
CONSORT diagram. P: Placebo; C: CBD. a: One was unavailable to attend Run 2 and one did not attend Run 2 due to illness; b: One was unavailable to attend Run 1 and one did not attend Run 1 due to injury; c: Withdrawn prior to treatment administration (no longer eligible due to illness); d: One was unavailable to attend Run 1 and two did not attend Run 1 due to illness; e: Did not attend Run 2 due to injury (sustained elsewhere); f: The untreated (ineligible) participant was excluded from the final sample

Fifty-five volunteers signed informed consent between July 17 and August 1, 2023, and 52 were randomised (Fig. 1). Of those randomised: (1) 43 received both treatments (i.e., as intended); (2) eight received one treatment (after failing to attend either the first [*n* = 5] or second [*n* = 3] treatment session); and (3) one was withdrawn prior to treatment administration. This individual was deemed no longer able (i.e., safe) to perform endurance exercise and excluded from the final (analytical) sample. The remaining 51 (randomised) participants were included in this sample.

#### Note

Recruitment ceased before the target sample size was met because the trial close date was fixed (i.e., the treatment sessions were booked in advance and could not be rescheduled) and fewer than 84 participants enrolled before this time.

### Participant Characteristics

The demographic characteristics of the participant population are summarised in Table 1. In general, the sample was young, had a body mass index in the ‘healthy’ range, and contained slightly more males (57%) than females (43%). Participants were typically active – but unaccustomed to running distances > 5 km. While most individuals (53%) had tried cannabis – few (*n* = 2; ∼4%) had used it in the last 4 weeks. Only four participants (∼ 8%) had ever tried CBD, and none had used it in the last 3 months.

**Table 1:** Baseline characteristics (*n* = 51)

Characteristic	Frequency (*n*), Mean (95% CI) or Median [IQR]
**General:**	
Sex (n) ^a^	
Male	29 (57%)
Female	22 (43%)
Females using hormonal contraceptives	10 (45%)
Age (years)	22 [21–25]
Height (m)	1.75 [1.68–1.83]
Weight (kg)	76.6 (73.1, 80.2)
BMI (kg/m^2^)	24.1 (22.7, 25.4)
**Recent Physical Activity**:	
Moderate in the last week (days)	2 [2–4]
Moderate in the last week (minutes/event) ^b^	60 [45–90]
Vigorous in the last week (days)	4 [3–5]
Vigorous in the last week (minutes/event) ^c^	60 [45–75]
**Recent Running**:	
Days in the last fortnight (n)	
Zero	10 (20%)
1–4 days	30 (59%)
5–9 days	9 (18%)
10–14 days	2 (4%)
Distance (km/event) ^d^	4.8 [3.0–5.0]
Duration (minutes/event) ^e^	36 (30–43)
**Cannabis Use**:	
Ever Used (n)	
No	24 (47%)
Yes	27 (53%)
Lifetime exposures (n) ^f^	
≤ 10 uses	16 (31%)
> 10 uses	10 (20%)
Time since last use (n)	
≤ 1 week	1 (2%)
1–4 weeks	1 (2%)
1–3 months	3 (6%)
3–6 months	4 (8%)
6–12 months	5 (10%)
> 1 year	13 (25%)
Reason(s) for use (all) (n)	
Recreational purposes	25
Medicinal purposes	1
General health and wellbeing	2
Route(s) of administration (all) (n)	
Inhalation	25
Oral ingestion	7
Topical	0
**CBD Use**:	
Ever Used (n)	
No	47 (92%)
Yes	4 (8%)
Lifetime exposures (n)	
≤ 10 uses	3 (6%)
> 10 uses	1 (2%)
Time since last use (n)	
≤ 1 week	0
1–4 weeks	0
1–3 months	0
3–6 months	2 (4%)
6–12 months	1 (2%)
> 1 year	1 (2%)
Reason(s) for use (all) (n)	
Recreational purposes	2
Medicinal purposes	1
General health and wellbeing	1
Route(s) of administration (all) (n)	
Inhalation	2
Oral ingestion	2
Topical	1
**Expectations**:	
Effect of CBD on exercise performance (n)	
Very Negative	0
Slightly Negative	1 (2%)
No Effect	16 (31%)
Slightly Positive	24 (47%)
Very Positive	2 (4%)
I don’t know	8 (16%)
Effect of CBD on exercise enjoyment (n)	
Very Negative	0
Slightly Negative	0
No Effect	8 (16%)
Slightly Positive	33 (65%)
Very Positive	5 (10%)
I don’t know	5 (10%)
Overall ‘feeling’ about running 10 km ^g^	50 [38–82]
Perceived difficulty of running 10 km ^h^	66 [50–75]

Values are frequency (n), mean (95% CI) or median [IQR], as appropriate (i.e., where data are normal and non-normal, respectively). BMI: Body Mass Index; CI: Confidence Interval; IQR: Interquartile Range; a: All males identified as ‘men’ and all females identified as ‘women’; b: Excludes eight ‘unknowns’ (and six participants who did not do moderate exercise); c: Excludes three ‘unknowns’ (and two participants who did not do vigorous exercise); d: Excludes seven ‘unknowns’; e: Excludes two ‘unknowns’; f: Excludes one ‘unknown’; g: On a 100 mm visual analog scale (VAS), where zero = “Negative” and 100 = “Positive”; h: On a 100 mm VAS, where zero = “Not at all” and 100 = “Extremely”

### Standardisation Procedures

The following (minor) non-compliances were noted: (1) two instances of failure to avoid exercise (both involving the same participant); (2) two instances of failure to spend ≥ 6 h in bed (both ≥ 5 h; one per treatment); and (3) two instances of caffeine being consumed (a) ≤ 45 min prior to arrival (20 and 28 min; one per treatment) and (b) prior to one treatment session, only (both on placebo).

Sleep duration, sleep quality, hydration status (i.e., urine specific gravity), the time-of-day participants commenced exercise (‘start time’) and the length of time between caffeine use and exercise (‘caffeine use’) did not differ significantly by Treatment, though start time (Run 1: 9:24 (9:17, 9:31) AM; Run 2: 9:08 (9:01, 9:15) AM, *p* < 0.001) and caffeine use (Run 1: 206 (193, 220) minutes; Run 2: 187 (173, 200) minutes, *p* = 0.011) differed by Run (Table 2) (as participants arrived at Run 2 earlier than Run 1).

**Table 2:** Descriptive statistics

	Placebo (Mean (95% CI) or Median [IQR])	CBD (Mean (95% CI) or Median [IQR])	Effect Size (Cohen’s d_rm_^a^)
**Primary Outcomes**			
**Affective Valence**_(scale: -5–5)_			
Lap 6	2.0 [0.0–2.0]	1.0 [0.0–2.0]	-0.14
Lap 12	0.0 [-1.0–1.0]	0.0 [-2.0–2.0]	-0.20
Lap 18	-1.0 [-2.0–1.0]	-1.0 [-2.0–1.0]	-0.06
Lap 24	-1.0 [-3.0–1.0]	-1.0 [-2.8–1.0]	-0.03
**Positive Affect**_(scale: 10–50)_			
Baseline	24.8 (22.8, 26.8)	25.8 (23.7, 27.9)	0.08
Pre-Run	24.4 (22.4, 26.3)	23.8 (21.4, 26.1)	-0.10
Post-Run	26.2 (23.9, 28.4)	26.0 (23.8, 28.1)	0.01
**Negative Affect**_(scale: 10–50)_			
Baseline	12.0 [10.0–14.3]	12.0 [11.0–13.0]	0.04
Pre-Run	11.5 [10.0–13.0]	11.0 [10.0–13.0]	0.01
Post-Run	10.0 [10.0–11.0]	10.0 [10.0–11.8]	0.06
**Secondary Outcomes**			
**Exercise Enjoyment**_(scale: 18–126)_			
Post-Run	78.6 (74.6, 82.6)	78.2 (74.2, 82.1)	-0.14
**Exercise Motivation**_(0–100 mm)_			
Post-Run	54 [34–75]	61 [36–67]	-0.11
**Exercise Self-Efficacy**_(0–100 mm)_			
Post-Run	79 [65–99]	74 [57–99]	-0.06
**Euphoria**_(0–100 mm)_			
Baseline	31 [20–50]	40 [4–50]	-0.03
Pre-Run	28 [15–50]	34 [0–50]	-0.15
Post-Run	28 [14–62]	36 [8–54]	0.01
**Pain**_(0–100 mm)_			
Baseline	13 [0–30]	3 [0–20]	-0.22
Pre-Run	2 [0–25]	2 [0–28]	-0.01
Post-Run	32 [5–62]	32 [8–62]	0.04
**Anxiety**_(0–100 mm)_			
Baseline	24 [0–50]	20 [4–36]	-0.03
Pre-Run	28 [6–43]	20 [0–50]	0.04
Post-Run	1 [0–8]	0 [0–50]	0.18
**Sedation**_(0–100 mm)_			
Baseline	13 [0–25]	11 [0–29]	-0.06
Pre-Run	12 [0–30]	15 [0–25]	-0.08
Post-Run	6 [0–35]	13 [0–29]	-0.05
**Perceived Exertion**_(scale: 6–20)_			
Lap 6	12.0 [11.0–13.0]	12.0 [10.0–13.0]	-0.04
Lap 12	13.0 [12.0–14.0]	13.0 [12.0–14.0]	-0.16
Lap 18	14.5 [12.0–15.3]	13.5 [12.0–15.0]	-0.16
Lap 24	15.0 [13.0–17.0]	14.0 [12.3–16.0]	-0.21
Run Time (minutes)	65.8 (62.7, 68.9)	66.8 (63.8, 69.7)	0.13
**Standardisation Variables**			
Sleep Duration (hours)	7.0 [6.5–7.5]	7.0 [6.6–8.0]	-
Sleep Quality ^b^	4 [3–4]	4 [3–4]	-
Urine Specific Gravity	1.009 [1.004–1.017]	1.012 [1.003–1.019]	-
Caffeine Use (minutes) ^c^	195 (181, 209)	198 (185, 212)	-
Start Time (AM) ^d^	9:14 [8:58–9:38]	9:14 [8:56–9:39]	-

Values are mean (95% CI) or median [IQR], as appropriate (i.e., where the data are normal and non-normal, respectively). CI: Confidence Interval; IQR: Interquartile Range. a: Cohen’s d_rm_ was calculated as described elsewhere [62] using paired means and standard deviations, only (i.e., participants without missing data) – note that positive values signify an increase (i.e., from placebo) on CBD; b: Where 1 = ‘Very Bad’, 2 = ‘Fairly Bad’, 3 = ‘Neither Bad nor Good’, 4 = ‘Fairly Good’ and 5 = ‘Very Good’; c: The length of time between caffeine use and exercise, where caffeine was consumed (*n* = 25); d: The time-of-day participants commenced exercise. Note the following missing data: Two instances of failure to complete the pre-run questionnaire (both on CBD) and one instance of failure to complete the post-run questionnaire (on placebo)

Both treatment sessions were conducted under similar environmental conditions (∼ 18 °C) (Table S1) with most participants running *predominantly alone* (*n* = 41) and either *as fast as comfortably possible* (*n* = 25) or *at a tolerable pace* (*n* = 22).

### Primary Outcomes

**Fig. 2:**
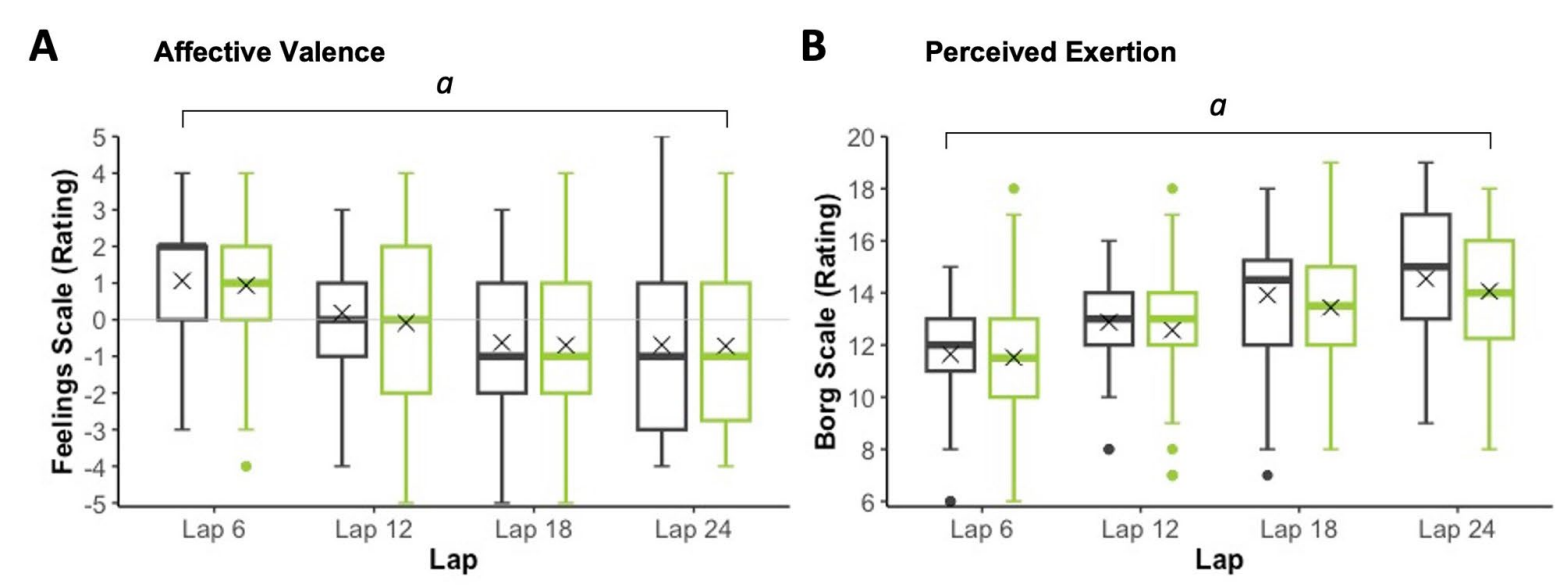
Affective Valence (**A**) and Perceived Exertion (**B**) during exercise. **Grey**: Placebo; **Green**: 150 mg CBD. ‘*X*’ represents the mean value. *a*: Differs by Lap and Lap^2^ (*p*’s < 0.05)

Affective valence (FS) did not demonstrate a significant main effect of Treatment or a Treatment × Lap interaction (Tables 2 and 3; Fig. 2). A priori planned post hoc comparisons of FS ratings on placebo and CBD at laps 6 (*p* = 0.395), 12 (*p* = 0.442), 18 (*p* = 0.660) and 24 (*p* = 0.927) likewise found no differences between the treatments. To ‘verify’ this lack of effect (i.e., determine whether there was truly no effect or if these non-significant results were due to the study being underpowered), we calculated the 95% CI around the Cohen’s d_z_ effect of CBD on affective valence at each time point (i.e., lap) [8] (Note: FS ratings were treated as continuous and only paired data could be included; *n* = 43). The ‘target’ Cohen’s *d*_z_ effect of 0.40 (as defined in Sect. 2.8.1) did not fall within the calculated 95% CI on laps 6 (-0.44, 0.16), 12 (-0.47, 0.10), 18 (-0.37, 0.24) or 24 (-0.36, 0.29). Thus, the likelihood of CBD having this effect (even in a larger participant population) appears low.

**Table 3:** The probability (*p*) values for the fixed effects included in each statistical analysis

	Model	Fixed Effects (Probability Values)							
		Treatment	Run	Sex	Time	Treatment × Time	Lap	Lap^2^	Treatment × Lap
**Primary Outcomes**:									
Affective Valence	Standard	0.522	NR	**0.026**	-	-	**< 0.001**	**0.002**	0.576
Positive Affect	Gamma (1)	0.935	NR	NR	**0.001**	0.332	-	-	-
Negative Affect	Gamma (2)	0.665	**< 0.001**	NR	**< 0.001**	0.652	-	-	-
**Secondary Outcomes**:									
Exercise Enjoyment	Standard	0.634	0.517	0.280	-	-	-	-	-
Exercise Motivation	Standard	0.557	0.817	**0.005**	-	-	-	-	-
Exercise Self-Efficacy	Gamma (2)	0.503	NR	NR	-	-	-	-	-
Euphoria ^a^	Standard	0.550	0.512	0.373	0.282	0.826	-	-	-
Pain	LOG	0.778	NR	NR	**< 0.001**	0.413	-	-	-
Anxiety	LOG	0.905	NR	0.116	**< 0.001**	0.232	-	-	-
Sedation	Gamma (2)	0.200	NR	NR	0.421	0.755	-	-	-
Perceived Exertion	Standard	0.149	0.111	NR	-	-	**< 0.001**	**0.049**	0.451
Run Time	SQRT	0.195	NR	**0.017**	-	-	-	-	-
**Standardisation Variables**:									
Sleep Duration	Standard	0.579	NR	NR	-	-	-	-	-
Sleep Quality	Standard	0.925	NR	NR	-	-	-	-	-
Urine Specific Gravity	Gamma (2)	0.513	NR	**0.042** ^** d**^	-	-	-	-	-
Caffeine Use ^b^	Standard	0.960	**0.011**	0.205	-	-	-	-	-
Start Time ^c^	Standard	0.583	**< 0.001**	0.568	-	-	-	-	-

-: Not Applicable; Gamma (1): The gamma model used an identity link; Gamma (2): The gamma model used a log link; LOG: The dependent variable was log transformed; NR: Not Required (i.e., did not reduce the AIC value of the model); SQRT: The dependent variable was square-root transformed. a: The ‘best’ possible model (Shapiro-Wilk, *p* > 0.05; Levene, *p* = 0.002); b: The length of time between caffeine use and exercise, where caffeine was consumed (*n* = 25); c: The time-of-day participants commenced exercise; d: Males had higher urine specific gravities than Females 1.013 (1.010, 1.016) vs. 1.010 (1.00, 1.011)

**Fig. 3:**
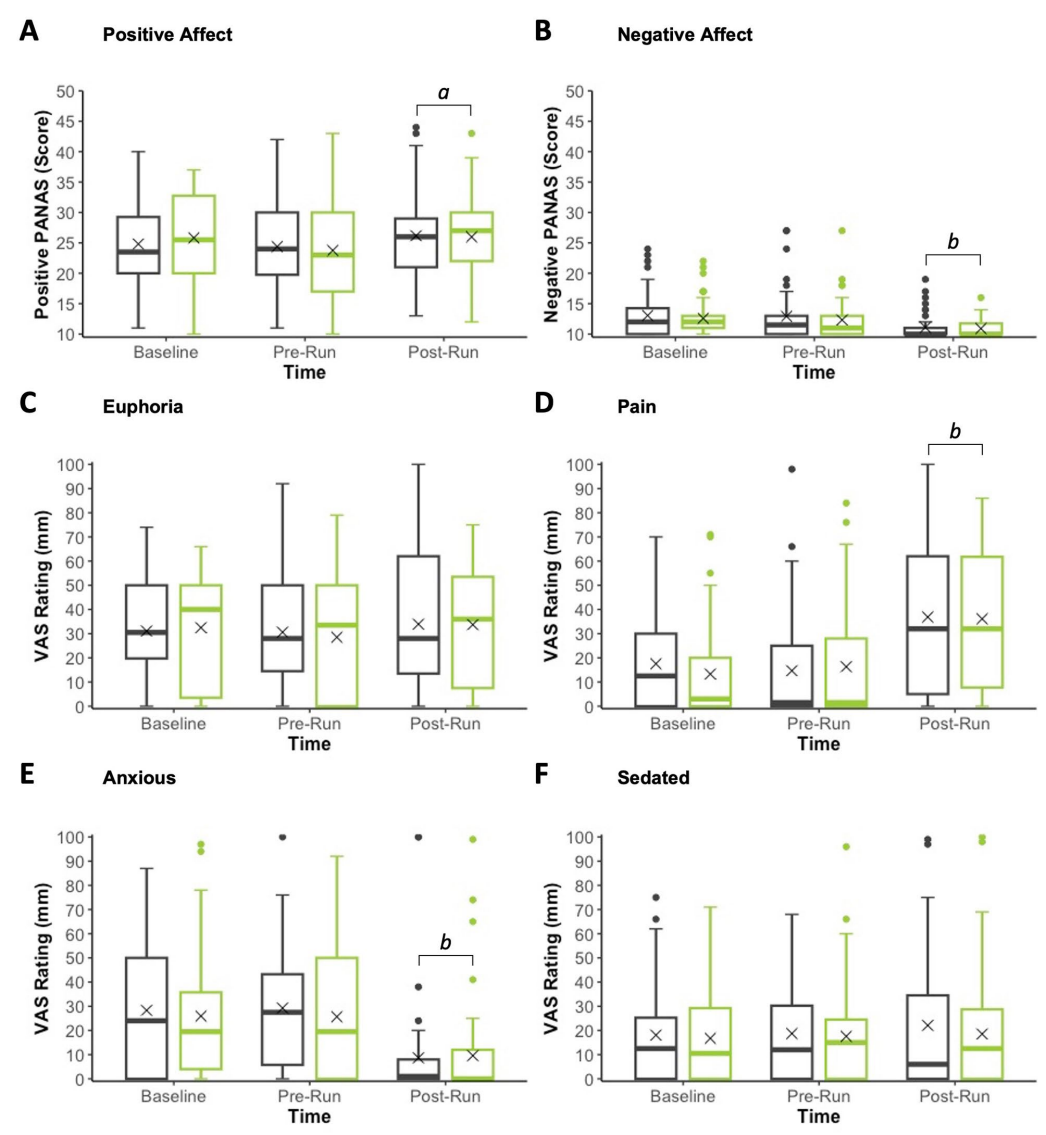
Positive Affect (**A**), Negative Affect (**B**), Euphoria (**C**), Pain (**D**), Anxious (**E**) and Sedated (**F**) at Baseline (i.e., pre-treatment), Pre-Run and Post-Run. **Grey**: Placebo; **Green**: 150 mg CBD. ‘*X*’ represents the mean value. *a*: Differs from Pre-Run (*p* < 0.05); and *b*: Differs from Pre-Run and Baseline (*p*’s < 0.05). PANAS: Positive and Negative Affect Schedule; VAS: Visual Analog Scale

Neither positive nor negative affect (PANAS) demonstrated a significant main effect of Treatment or a Treatment × Time interaction (Tables 2 and 3; Fig. 3).

### Secondary Outcomes

**Fig. 4:**
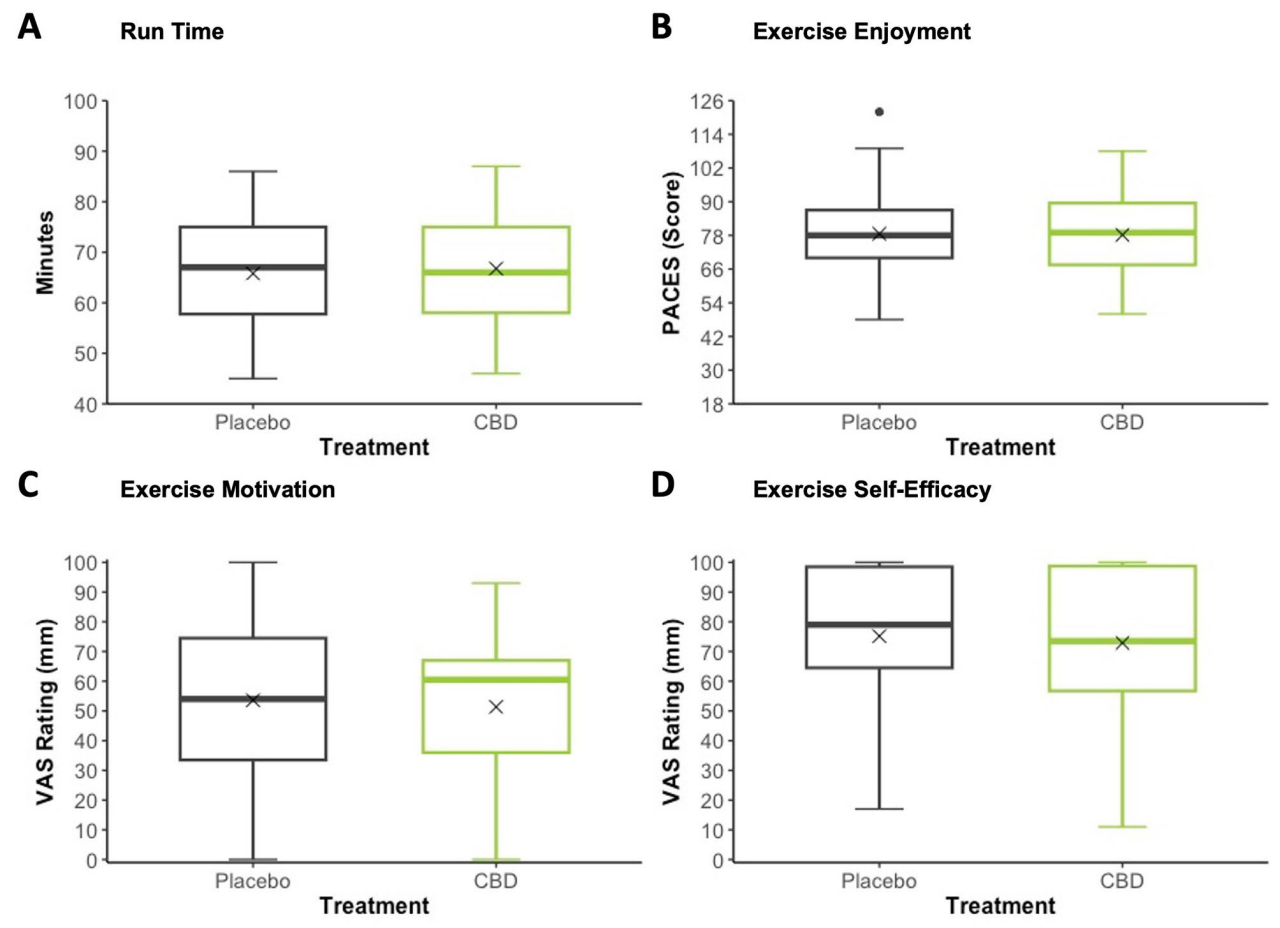
Run Time (**A**), Exercise Enjoyment (**B**), Exercise Motivation (**C**), Exercise Self-Efficacy (**D**) (all assessed Post-Run). **Grey**: Placebo; **Green**: 150 mg CBD. ‘*X*’ represents the mean value. PACES: Physical Activity Enjoyment Scale; VAS: Visual Analog Scale

None of the secondary outcomes measured demonstrated significant main effects of Treatment or Treatment × Time (or Lap) interactions (Tables 2 and 3; Figs. 3 and 4).

### Tertiary Observations

Affective valence, exercise motivation and run time all demonstrated main effects of Sex (Table 3), with females: (1) reporting more positive affect during exercise (0.0 (-0.8, 0.9) vs. -1.1 (-1.9, -0.4), *p* = 0.026); (2) reporting greater motivation to undertake further exercise (62 (53, 70) vs. 46 (38, 53) mm, *p* = 0.005); and (3) running more slowly (69.7 (65.4, 74.0) vs. 62.9 (59.3, 66.4) minutes, *p* = 0.017) than males.

Positive and negative affect, pain, and anxiety all demonstrated main effects of Time (Table 3). Both positive affect and pain were increased post-run (27.5 (25.2, 29.8) and 18 (12, 27) mm, respectively) compared to pre-run (25.4 (23.1, 27.8), *p* < 0.001 and 5 (3, 7) mm, *p* < 0.001, respectively) and baseline (26.7 (24.3, 29.0), *p* = 0.051 and (6 (4, 9) mm, *p* < 0.001, respectively)– while both negative affect and anxiety were decreased post-run (11.0 (10.3, 11.7) and 3 (2, 5) mm, respectively) compared to pre-run (12.3 (11.5, 13.2), *p* < 0.001 and 12 (8, 18) mm, *p* < 0.001, respectively) and baseline (12.6 (11.7, 13.5), *p* < 0.001 and 12 (8, 18) mm, *p* < 0.001, respectively).

Negative affect demonstrated a main effect of Run (i.e., trial order) (Table 3), with higher scores (i.e., more negative affect) observed on Run 1 than Run 2 (12.6 (11.7, 13.5) vs. 11.2 (10.5, 12.1), *p* < 0.001).

Affective valence and perceived exertion demonstrated main effects of Lap and Lap^2^ (Table 3), with the former decreasing and the latter increasing throughout exercise.

### Blinding and Adverse Events

48% of participants (*n* = 23/48) believed they received placebo on placebo and 39% of participants (*n* = 18/46) believed they received CBD on CBD. The remainder incorrectly guessed the opposing treatment.

Seven adverse events (all mild) were reported post-run (i.e., post-treatment administration); three following placebo (fatigue, knee pain, dizziness) and four following CBD (fatigue, muscle cramp, stiff muscles, sore Iliotibial band).

## Discussion

This study investigated the effects of CBD on subjective responses to self-paced endurance exercise in recreationally active individuals. Contrary to our hypothesis, it showed that CBD (150 mg, oral) did not alter affective valence during or following exercise (i.e., a ∼ 10 km run). Other subjective feelings (i.e., enjoyment, motivation, self-efficacy, euphoria, pain, anxiety, sedation) were likewise unchanged.

Two previous studies have investigated the effects of CBD on subjective responses to exercise [6, 29]. Both found that CBD *increased* positive affect. However, one [6] was unblinded and co-administered a low, but not negligible, dose of THC (∼ 4 mg). The other [29] used: (1) a 300 mg dose of CBD; (2) a fixed-intensity treadmill run; and (3) an endurance-trained population. These methodological features likely increased its sensitivity (i.e., to change) compared to the current investigation, which used a lower 150 mg dose, self-paced outdoor run, and recreationally active population. However, they also limit the study’s ecological validity. Indeed, while doses ≥ 300 mg have demonstrated more consistent therapeutic effects [11], they are less readily available [11, 12]. Likewise, although fixed-intensity exercise produces less varied/noisy responses, endurance exercise is often self-paced. (The endurance-trained population is also much smaller than the recreationally active one). Put simply, our findings suggest that the subjective effects of CBD observed in prior studies [6, 29] might not be sustained under typical ‘real-world’ conditions. Further research is required to determine if CBD is efficacious, and if so, which condition/s (e.g., dose, ‘type’ of exercise, participant population) is/are moderating its effects.

Neither this study nor either of those published previously observed an effect of CBD on perceived exertion or ‘run time’ [6, 29]. This, along with the finding that CBD does not compromise the subjective experience of exercise, suggests it is unlikely to impede physical activity participation, which is significant given its apparent popularity [50]. Indeed, CBD use (e.g., for medicinal and/or ‘wellness’ purposes) has become common in North America and Europe where products can be purchased online and over-the-counter [11, 12]. Two recent randomised, placebo-controlled trials [51, 52] likewise found no effect of chronic CBD use on physical activity participation in healthy free-living adults – albeit at very low doses (10 and 50 mg/day).

Finally, it is worthwhile noting that, although well-established [53], this study elegantly demonstrates the powerful mood-enhancing effects of exercise. Indeed, despite inducing negative affect and pain, the 10 km runs, once completed, increased positive affect, and decreased negative affect and anxiety: effects that have previously been attributed, in part, to endogenous cannabinoids (e.g., anandamide) [53].

One strength this study has over others in its field [5, 6, 29] is that it was able to investigate the effects of CBD in the presence (on average) of *negative affect* (i.e., negative rather than positive FS ratings; see Table 2). A second strength is that it measured affect *during* (not just following, e.g. [5]), exercise. Indeed, ‘in-exercise’ measures more reliably predict future physical activity participation [54, 55].

This study is, however, limited in several aspects:

First, no physiological or biochemical (e.g., plasma CBD concentration) measures were taken. Indeed, these were impractical to obtain ‘en masse’. The few studies that have investigated the effects of CBD on exercise physiology suggest it has either no effect (∼ 13.6 mg, inhaled) [56] or a ‘possible’ effect to increase VO_2_ and VO_2max_ (300 mg, oral) [29] at fixed-intensities. The pharmacokinetics of the soft-gel capsules used in this investigation have been characterised (by the supplier) [57] – but not yet publicly disclosed.

Second, no habituation session was conducted. Indeed, we were concerned that the addition of a *third* 10 km run might deter some individuals (particularly those most likely to experience negative affect during exercise) from participating. In the end, however, only one outcome measure (i.e., negative affect) demonstrated a significant main effect of Run (i.e., trial order) – and this was handled analytically.

Third, we were unable to standardise menstrual phase. That said, there is relatively limited evidence that menstrual phase influences subjective responses to exercise (i.e., the effects reported to date appear inconsistent and sporadic) [58–60]. It should also be noted that the current investigation was designed to determine whether the subjective effects of CBD observed in prior studies were sustained under more ecologically valid conditions; that is, in the presence of ‘real-world’ factors such as this.

Fourth, participants *could* have used external CBD in the 7 days preceding Run 1, and/or between Run 1 and Run 2, as abstinence was only verified 24 h prior to each treatment session. That said, as: (1) only four participants (∼ 8%) had ever tried CBD (Table 1); (2) none had used it in the last 3 months (Table 1); and (3) CBD cannot (yet) be accessed without a prescription in Australia [12], this seems reasonably unlikely.

Fifth, running pace was not measured. This could have been altered, even though total run time was not.

Finally, it should be noted that although synthetic -(-) CBD is chemically identical to plant-derived CBD, plant-derived CBD *products* often contain additional cannabinoids and cannabis constituents that are lacking in synthetic ones (such as ours). These constituents are usually only present in low concentrations. However, their inclusion does mean that plant-derived CBD products have the potential to produce slightly different effects [61].

## Conclusion

CBD, taken at the relatively low oral dose of 150 mg, does not appear to enhance the subjective experience of self-paced endurance exercise in recreationally active individuals. Nor, however, does it appear to compromise it. These findings suggest that CBD use is safe under exercise conditions and unlikely to impede physical activity participation, which is significant given its apparent popularity. Our study also reaffirms the powerful mood-enhancing effects of exercise.

### Electronic Supplementary Material

Below is the link to the electronic supplementary material.

Supplementary Material 1

## Data Availability

The deidentified participant data are available from Dr Danielle McCartney upon reasonable request (danielle.mccartney@sydney.edu.au).

## Declarations

### HREC Approval

The study was approved by Griffith University’s Human Research Ethics Committee (GU Ref No: 2023/253) and performed in accordance with the standards of ethics outlined in the Declaration of Helsinki.
