# Supplementary material for: The Effect of Cannabidiol on Subjective Responses to Endurance Exercise: A Randomised Controlled Trial
Source: Sports Med Open. 2024 May 23;10:61. doi: 10.1186/s40798-024-00727-3 (PMC11116295; doi:10.1186/s40798-024-00727-3)
Supplement: Supplementary file 1 — Supplementary Material 1 [file 40798_2024_727_MOESM1_ESM.pdf]

SUPPLEMENTARY FILE 1

JOURNAL: **Sports Medicine – Open**

ARTICLE: **The effect of cannabidiol on subjective responses to endurance exercise: A randomised controlled trial.**

AUTHORS: Danielle McCartney[1, 2, 3], Christopher Irwin[4, 5], Zeeta Bawa[1, 2, 6], Blake Palmer[4], Ayshe Sahinovic[1, 2, 3], Nathan Delang[4], Gregory R. Cox[7], Ben Desbrow[4], Namson S. Lau[8] & Iain McGregor[1, 2, 3].

AFFILIATIONS:

[1] Lambert Initiative for Cannabinoid Therapeutics, The University of Sydney.
[2] Brain and Mind Centre, The University of Sydney.
[3] School of Psychology, Faculty of Science, The University of Sydney.
[4] School of Health Sciences and Social Work, Griffith University.
[5] Menzies Health Institute Queensland, Griffith University.
[6] Sydney Pharmacy School, The University of Sydney.
[7] Faculty of Health Sciences and Medicine, Bond University.
[8] The Boden Initiative, Charles Perkins Centre, The University of Sydney.

Table S1. Environmental conditions in Southport, Queensland

	**Run 1** (August 21, 2023)	**Run 2** (August 28, 2023)
**Temperature (°C)**		
8:00 AM	16	17
9:00 AM	18	18
10:00 AM	19	19
11:00 AM	20	19
12:00 PM	20	19
**Humidity (%)**		
8:00 AM	63	75
9:00 AM	59	71
10:00 AM	57	68
11:00 AM	56	66
12:00 PM	55	68
**Description**		
8:00 AM	Sunny	Partly Cloudy
9:00 AM	Sunny	Sunny
10:00 AM	Sunny	Sunny
11:00 AM	Sunny	Sunny
12:00 PM	Sunny	Sunny
**Wind Speed (km/h)**		
8:00 AM	6	9
9:00 AM	4	10
10:00 AM	4	11
11:00 AM	6	12
12:00 PM	8	13
**Wind Direction**		
8:00 AM	W	S
9:00 AM	WNW	SSE
10:00 AM	N	SE
11:00 AM	NNE	SE
12:00 PM	NE	SE
**Precipitation (mm)**		
8:00 AM–12:00 PM	0.0	0.0

Information obtained from ‘World Weather Online’. E: East; N: North; S: South; W: West.
